# Women’s Pain Management Across the Lifespan—A Narrative Review of Hormonal, Physiological, and Psychosocial Perspectives

**DOI:** 10.3390/jcm14103427

**Published:** 2025-05-14

**Authors:** Andrea Stieger, Auste Asadauskas, Markus M. Luedi, Lukas Andereggen

**Affiliations:** 1 Department of Anaesthesiology, Rescue- and Pain Medicine, Cantonal Hospital of St. Gallen, 9007 St. Gallen, Switzerland; andrea.stieger@h-och.ch (A.S.);; 2 Department of Neurology, University Hospital of Bern, Inselspital Bern, 3010 Bern, Switzerland; 3 Department of Anaesthesiology and Pain Medicine, Inselspital, Bern University Hospital, University of Bern, 3010 Bern, Switzerland; 4 Department of Neurosurgery, Kantonsspital Aarau, 5001 Aarau, Switzerland; 5 Faculty of Medicine, University of Bern, 3010 Bern, Switzerland

**Keywords:** pain perception, sex differences in pain, gender bias, hormonal influence

## Abstract

**Introduction**: Pain perception and management differ by sex, with women frequently experiencing more acute and chronic pain and greater disability than men. Yet, anesthesiology and pain control often overlook the physiological, hormonal, and psychological factors shaping women’s pain. **Methods**: This narrative review explores essential considerations from current literature to optimize pain management in women. We examine evidence about the impact of hormonal changes, reproductive transitions, and psychosocial factors on pain perception and responses to analgesics. By highlighting key insights and practical strategies, we aim to support the development of personalized pain management approaches tailored to women’s unique needs. **Results**: Hormonal changes, including variations in estrogen and progesterone levels, significantly influence pain thresholds and the effectiveness of analgesics and anesthetics. The menstrual cycle, menopausal transitions, and pregnancy each alter pain responses, necessitating personalized approaches to pain management. Postmenopausal women are particularly vulnerable to chronic pain conditions, such as those associated with osteoporosis, which require adjustments in long-term treatment strategies. Furthermore, psychosocial factors like anxiety and emotional distress can intensify pain, highlighting the need for holistic, integrative care. **Conclusions**: The existing gaps in women’s pain management across the lifespan highlight the need to revise both anesthesia and pain management protocols to better account for sex-specific biological and psychosocial factors. Addressing the unique biological and psychosocial factors that influence pain in women can enhance both the effectiveness and equity of care. By tailoring treatment strategies in women, clinicians can not only enhance pain management but also greatly improve their overall quality of life.

## 1. Introduction

Pain experiences and pain management are influenced by a complex interplay of physiological, hormonal, and psychological factors. Increasing evidence reveals significant sex differences in pain experiences, with women exhibiting a higher prevalence of both acute and chronic pain conditions, as well as greater levels of pain-related disability than men [1,2,3]. These disparities are attributed to both biological and psychosocial characteristics, including hormonal fluctuations, reproductive physiology [4,5], and emotional responses to pain [6,7]. Hormonal changes—particularly fluctuations in estrogen and progesterone levels throughout the menstrual cycle, pregnancy, and menopause—play a crucial role in modulating pain thresholds and influencing the effectiveness of pain-relieving treatments, including analgesics (which alleviate pain without affecting consciousness) and anesthetics (which block pain sensation, often by temporarily inducing loss of sensation or consciousness) [4,8,9]. Namely, estrogen can increase pain thresholds; however, reduced progesterone levels are associated with heightened pain sensitivity. As such, fluctuations in estrogen levels promote inflammation, which subsequently amplifies pain perception in females. In contrast, progesterone exerts protective effects by enhancing the expression of gamma-aminobutyric acid (GABA) in the brain [5].

Reproductive transitions, including the menstrual cycle, pregnancy, and menopause, further affect pain perception treatment response. For instance, hormonal shifts during the menstrual cycle can alter pain sensitivity and medication efficacy [5,8], while pregnancy presents unique challenges due to the contraindications of many pharmacological treatments [10]. In addition, postmenopausal women frequently experience chronic pain conditions, such as those associated with osteoporosis, requiring tailored adjustments in pain management strategies [5,11], as postmenopausal women often exhibit lower pain thresholds due to the decline in estrogen and progesterone levels [5].

Psychosocial factors add an additional layer of complexity, as women often experiencing higher levels of anxiety, depression, and emotional distress related to pain [6,12,13,14]. These factors not only influence the perception of pain but also affect the outcomes of pain management interventions. These factors can heighten the perception of pain through several mechanisms. For instance, anxiety and depression are associated with increased attentional focus on pain, reduced pain tolerance, and altered pain modulation pathways in the central nervous system. Emotional distress can amplify pain signals by increasing physiological arousal and reducing the effectiveness of endogenous pain inhibitory systems [15,16,17,18]. As a result, women may report more intense pain and respond differently to pain management interventions. Holistic care approaches that integrate both the biological and psychosocial dimensions of pain have shown potential in improving outcomes for women, yet their implementation remains inconsistent [19], often due to systemic barriers such as lack of interdisciplinary coordination, insufficient provider training, and limited healthcare resources [16,20]. Moreover, much of the existing literature focuses on general populations, with relatively few studies examining gender-specific adaptations or evaluating long-term outcomes in women [21]. This gap underscores the need for targeted research and practice models that address the unique biopsychosocial pain experiences of women, promoting more equitable and effective pain management [22].

Despite these differences, current anesthesiologic and pain management practices are often insufficiently tailored to address the distinct needs of women, leading to suboptimal outcomes and disparities in care [23,24].

This narrative review aims to identify the unique factors required for optimizing pain management in women. By focusing on hormonal influences, reproductive transitions, psychosocial factors, and current intervention strategies, we aim to highlight how acknowledging these variables can support more tailored treatments, ultimately contributing to the reduction of disparities by ensuring that women receive pain care that reflects their specific needs rather than a one-size-fits-all approach.

## 2. Differences in Pain Perception Between Men and Women

The epidemiology of women’s pain highlights the significant and often overlooked gender differences in pain prevalence, intensity, and treatment [25]. Research shows that women experience pain differently and more frequently than men, with higher prevalence rates of both acute and chronic pain conditions.

Research shows that women experience pain differently and more frequently than men, with higher prevalence rates of both acute and chronic pain conditions, which is thought to be influenced by biological factors such as hormonal fluctuations, genetic differences, and variations in pain processing pathways [1,26,27,28]. Additionally, sociocultural factors, such as gender roles and healthcare access, may contribute to these disparities [29,30].

According to the National Health Interview Survey (2019), 21.7% of women reported chronic pain compared to 19.0% of men. Additionally, high-impact chronic pain, significantly limiting life or work activities, was reported by 8.5% of women versus 6.3% of men [31]. A large-scale study across 17 countries further revealed that 45% of women experienced chronic pain, compared to 31% of men [32].

Women are disproportionately affected by conditions such as migraines, fibromyalgia, temporomandibular disorders, and irritable bowel syndrome [2,3]. For instance, women suffer from migraine up to three times more than men [33]. In addition, chronic pain disorders like osteoarthritis, rheumatoid arthritis, and neuropathic pain are more prevalent in women [1,29]. Studies also suggest that postoperative pain tends to be more intense in women than in men [1].

Some pain disorders are more prevalent in men, such as cluster headaches [3,34,35]. However, women are more likely to develop chronic pain following acute pain events, such as surgery or injury, highlighting sex-based differences in pain chronification mechanisms [36,37].

Mental health conditions also play a role in pain perception and chronicity. Women experience major depression at about twice the rate of men, with this difference emerging during adolescence and persisting throughout adulthood [12]. Anxiety disorders are similarly more prevalent in women and are often comorbid with chronic pain conditions [13].

Despite experiencing more frequent and intense pain, women often receive less aggressive pain treatment than men [21,38]. Gender bias in pain assessment and treatment means that women are more frequently prescribed sedatives and antidepressants rather than analgesics, leading to inadequate pain relief [39,40]. Understanding these epidemiological trends is crucial for developing sex-specific pain management strategies and ensuring equitable care for women.

## 3. Biological and Hormonal Factors in Women’s Pain Perception

**Figure 1:**
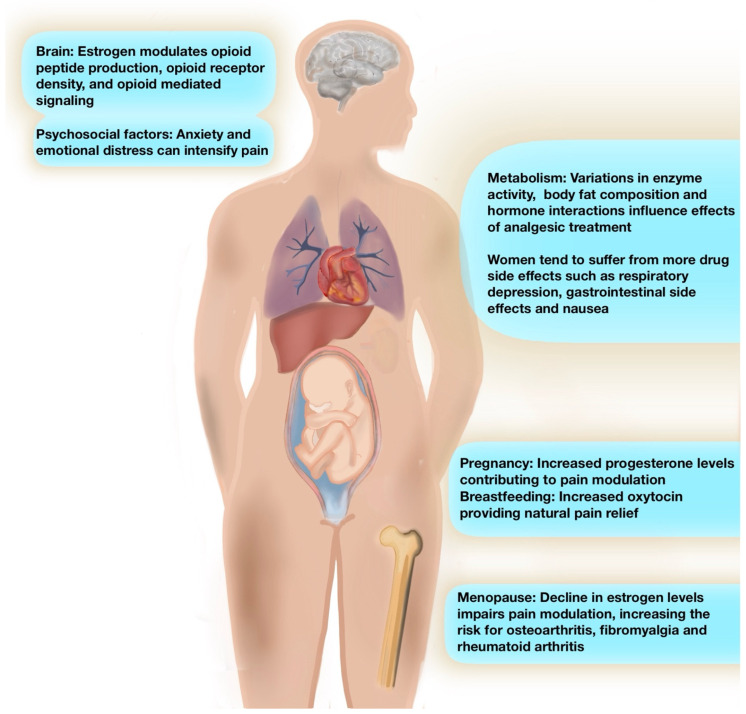
Conceptual overview of key factors influencing women’s pain across the lifespan. Figure legend: Summary of key physiological and psychosocial factors influencing pain perception in women across the lifespan. Hormonal shifts—such as estrogen’s modulation of opioid pathways—affect central pain processing. Psychosocial factors like anxiety and emotional distress can amplify pain. Metabolic differences influence analgesic response, with women more prone to side effects. Pregnancy-related progesterone increases support pain modulation, while estrogen decline during menopause impairs endogenous pain regulation.

Pain perception in women is influenced by hormonal and biological mechanisms [1,2,8,9,29,41]. This section explores hormonal changes across the menstrual cycle, pregnancy, breastfeeding, and menopause, emphasizing their impact on pain perception and treatment responses (Figure 1).

### 3.1. Hormonal Influence on Pain Modulation

#### 3.1.1. Estrogen, Progesterone, and Testosterone

Fluctuations in estrogen and progesterone play an important role in pain modulation. Estrogen has both pro-nociceptive and anti-nociceptive properties [42,43], meaning it can either increase or decrease pain sensitivity depending on receptor interactions. Estrogen enhances endogenous opioid function, modulates serotonin and dopamine pathways, and influences neural excitability, contributing to varying pain responses [8]. Meanwhile, progesterone is linked to increased inflammation and pain sensitivity, in particular during the luteal phase of the menstrual cycle [41]. In contrast, testosterone plays a crucial role in reducing pain perception. Research indicates that testosterone suppresses the production of pro-inflammatory cytokine, such as tumor necrosis factor α, contributing to reduced inflammation and pain sensitivity [5,44]. Furthermore, testosterone interacts with nociceptive pathways by decreasing capsaicin receptor-mediated signaling, reducing pain perception in dorsal root ganglion neurons [5]. Higher testosterone levels in men may help explain their lower prevalence of chronic pain conditions compared to women [5,9]; however, estrogen also plays a role in pain modulation in women, and at certain levels, it may exert analgesic effects similar to testosterone [1,4].

#### 3.1.2. Menstrual Cycle and Pain Sensitivity

The menstrual cycle influences pain sensitivity through hormonal fluctuations, with estrogen and progesterone playing pivotal roles in modulating pain perception at different phases. Fluctuations in estrogen and progesterone levels throughout the menstrual cycle significantly impact pain sensitivity. Studies suggest that pain thresholds vary depending on hormonal levels [5,45].

During the reproductive years, typically spanning from menarche (~age 12) to the onset of perimenopause (~mid-to-late 40s), these hormonal fluctuations follow a predictable cyclical pattern that profoundly affects pain thresholds [46].

During the follicular phase, rising estrogen levels promote endorphin release, which has an analgesic effect and improves pain tolerance by enhancing endogenous opioid function [8,9]. Estrogen also interacts with serotonin and dopamine pathways, further contributing to pain modulation and improved mood [47].

At ovulation, despite peak estrogen levels, some women report increased pain sensitivity [48]. This paradox occurs because high estrogen levels can also increase the expression of nociceptive (pain-related) receptors and amplify neural excitability [49].

In the luteal phase, progesterone levels rise while estrogen declines. The role of progesterone is complex. During pregnancy it helps regulate immune function and may reduce inflammation [50]. However, especially in women suffering from premenstrual syndrome or premenstrual dysphoric disorder progesterone withdrawal at the end triggers an inflammatory response increasing pain sensitivity and pro-inflammatory cytokines [41,51].

During the menstrual phase, both estrogen and progesterone levels are at their lowest, reducing the activation of endogenous pain-modulating systems and often leading to heightened pain perception [1].

#### 3.1.3. Cyclical Hormonal Conditions and Pain

Cyclical hormonal changes significantly impact pain perception and prevalence, influencing conditions such as menstrual migraine and temporomandibular disorder [3]. Estrogen withdrawal before menstruation is a known migraine trigger, and hormonal fluctuations play a key role in migraine pathogenesis [45]. These migraines typically affect women during the reproductive years, with symptoms often beginning after puberty and declining after menopause [52,53]. During the reproductive years, menstrual migraine affects around 4–8% of all women and around 20–25% of women with migraine [54]. However, other factors may contribute to migraine prevalence such as iron deficiency, which is more common in women, and which has been linked to increased frequency of migraines [55].

Research suggests that women with dysmenorrhea exhibit heightened sensitivity to deep muscle pain throughout the menstrual cycle, even in areas unrelated to menstrual pain [56]. Dysmenorrhea is most prevalent in adolescents and women in their twenties and thirties, correlating with peak reproductive hormone activity [57,58,59]. This indicates that pain sensitivity varies during menstruation due to structural and functional changes in the central nervous system. Variations in cerebral metabolism, pain-related brain activity, and gray matter volume in regions associated with pain modulation may contribute to this phenomenon [56,60,61]. These findings suggest that women with dysmenorrhea may have impaired pain inhibition and heightened pain facilitation, making them more sensitive to painful stimuli compared to women without the condition [62].

#### 3.1.4. Endometriosis and Chronic Pelvic Pain

Endometriosis is closely linked to chronic pelvic pain due to hormonal, inflammatory, and neural mechanisms. Endometriosis affects approximately 10% of women and is characterized by the growth of endometrial-like tissue outside the uterus. This condition leads to severe pelvic pain, dysmenorrhea, and potential infertility. Around 30% of affected women develop chronic pelvic pain that is unaffected by standard treatments [63].

Endometriosis often begins during adolescence or early adulthood, with symptoms intensifying during the reproductive years and sometimes subsiding after menopause due to reduced estrogen production [64,65]. Several mechanisms contribute to pain in endometriosis. Increased estrogen production due to increased aromatase activity may promote inflammation and nerve sensitization [66]. In addition, neurogenic inflammation may be exacerbated by the overexpression of vascular endothelial growth factor and nerve growth factor [67]. Altered pain processing in the central nervous system, particularly through enhanced glutamatergic neurotransmission and increased connectivity in pain-related brain regions, may further amplify pain perception [63]. Moreover, cross-organ sensitization links endometriosis to conditions such as irritable bowel syndrome, compounding the overall pain experience [68].

#### 3.1.5. Considerations During Pregnancy and Breastfeeding

Pregnancy introduces significant hormonal, mechanical, and physiological changes that influence pain perception and management. One of the key hormonal shifts involves increased progesterone levels, which acts as an anti-inflammatory agent by reducing immune responses and promoting the relaxation of smooth muscles [50]. This effect can contribute to pain modulation by mediating antinociception during pregnancy [69,70]. However, the impact of progesterone is context-dependent; while it reduces systemic inflammation, it may also cause joint laxity and musculoskeletal pain, increasing the risk of conditions such as pelvic girdle pain and lower back pain, as well as carpal tunnel syndrome [10,71]. These changes are specific to the reproductive years, with significant alterations during each trimester that uniquely affect pain thresholds [72,73,74].

Breastfeeding also introduces hormonal changes that affect pain sensitivity and require careful pain management strategies. A key physiological change during breastfeeding is the release of oxytocin, which has analgesic properties and may provide some natural pain relief. However, oxytocin surges can also induce uterine contractions, leading to postpartum cramping and discomfort [75]. Additionally, many breastfeeding women also experience postpartum musculoskeletal pain, particularly in the shoulders, neck, and back, often due to improper posture while nursing. Implementing ergonomic support, physical therapy, and stretching exercises can help alleviate these discomforts [76].

#### 3.1.6. Menopause and Chronic Pain

Menopause, typically occurring between the ages of 45 and 55, marks the permanent cessation of reproductive hormonal cycles and the transition to a state of sustained estrogen deficiency. This hormonal shift leads to significant physiological changes that can alter pain perception and increase vulnerability to chronic pain conditions. A key factor is the decline in estrogen levels, which impairs the body’s ability to modulate pain and has been associated with a heightened risk of musculoskeletal discomfort, particularly in the joints and lower back [70,77,78,79,80].

It may induce muscle apoptosis and lead to sarcopenia [79], as well as to bone loss leading to osteoporosis [69]. The decrease in estrogen is particularly associated with a higher risk of osteoarthritis, fibromyalgia, and rheumatoid arthritis [5,67,70]. Research also indicate that estrogen plays a key role in spinal health, and its decline accelerates intervertebral disc degeneration and osteoarthritis progression [11]. Postmenopausal women are twice as likely as men to experience low back pain. This underscores the impact of hormonal changes on chronic pain conditions [11].

Furthermore, vasomotor symptoms such as hot flushes and night sweats can disrupt sleep, potentially increasing pain perception [69]. A decline in endogenous opioids further reduces the body’s natural pain relief mechanisms, increasing the demand for pharmacological pain management [29].

### 3.2. Other Factors: Opioid Receptor Function and Immune Responses

#### 3.2.1. Opioid Sensitivity and Hormonal Influence

Estrogen significantly influences pain perception and opioid efficacy. Research suggests that estrogen modulates opioid peptide production, opioid receptor density, and opioid-mediated signaling in the brain [4]. Smith et al. describes an effect of high estrogen levels, enhancing mu-opioid receptor binding and resulting in greater activation of endogenous opioid neurotransmission and therefore resulting in a reduction of pain sensitivity [81]. However, evidence regarding the interactions between estrogen and opioid receptors remains limited to date [82]. Understanding these interactions could have critical implications for pain management, particularly in menopausal women or those using hormonal contraceptives.

#### 3.2.2. Sex Differences in Immune-Mediated Pain Responses

Estrogen plays a crucial role in regulating the immune system and modulating inflammatory responses, contributing to differences in pain processing between sexes. It enhances a stronger anti-inflammatory response by promoting a Th2 cell dominant immune profile. This results in a better response to vaccines and infections and may explain the higher prevalence of autoimmune disorders in females compared to males [83,84]. Research also indicates sex-based differences in tumor immunity. However, the hormonal interactions are complex’ and their influence remains unclear to date [85].

Furthermore, male macrophages tend to be more active than in females, contributing to peripheral pain generation. In addition, microglial cells, which play a role in central pain signaling, exhibit greater activity in males, while in females, they demonstrate higher phagocytic activity. This suggests that immune-related pain pathways differ between the sexes, with unique mechanisms influencing pain perception [5].

While research indicates that estrogen has immunostimulatory roles, progesterone and androgens are, in general, considered immunosuppressive. Lower testosterone levels are associated with an increase in B cells and therefore enhanced antibody release. As such, testosterone promotes an increased Th1 response, which down-regulates natural killer cells and tumor necrosis factor-alpha [83]. During menopause, changes in sex hormone levels lead to an imbalance in the immune response [83].

## 4. Psychological Factors and Pain Perception

Psychological factors, such as anxiety and depression, play a crucial role in pain perception and chronic pain conditions, with women experiencing higher prevalence rates than men. Namely, women are twice as likely as men to develop major depressive disorder and generalized anxiety disorder [6,12]. In chronic pain populations, the prevalence of depression and anxiety occurs in around 40% of patients, with women being disproportionately affected [14].

Several socioeconomic factors, including being unmarried, having a lower level of education, and earning a lower per capita monthly income, are significantly associated with an increased risk of depression and anxiety [86]. Anxiety can heighten pain perception by amplifying the body’s stress response, leading to greater activation of the hypothalamic-pituitary-adrenal axis [87]. In chronic pain conditions, both anxiety and depression contribute to pain chronification by disrupting neurotransmitter balance [88], reducing endogenous pain inhibition [89], and increasing central sensitization [90].

Women with chronic pain disorders such as fibromyalgia, irritable bowel syndrome, and temporomandibular disorders, exhibit higher rates of depression and anxiety. This highlights the bidirectional relationship between pain and mental health [14].

Several factors contribute to the increased prevalence of anxiety and depression in women. Changes in hormones like estrogen and progesterone can affect neurotransmitters like serotonin and dopamine, which may increase vulnerability to mood disorders. Furthermore, women often face added stress from social and psychological stressors such as caregiving burdens, trauma exposure, and discrimination, which may further exacerbate psychological distress and pain vulnerability [7,91]. Women also tend to engage in greater pain-related reflection and catastrophizing, which heightens perceived pain intensity and prolongs chronic pain experiences [92].

## 5. Gender Bias in Diagnosis and Treatment of Pain-Related Conditions

### 5.1. Diagnostic Bias and Pain Perception

Women’s pain is often overlooked or attributed to psychological causes rather than underlying physical conditions, resulting in delayed diagnoses and inadequate treatment [91]. A systematic review of 77 studies found that women’s pain is frequently downplayed as emotional or exaggerated, whereas men’s pain is taken more seriously and treated promptly [21]. This bias is especially evident in conditions like endometriosis, which often remains underdiagnosed [67]. Consequently, women with autoimmune diseases may experience misdiagnoses, and those with endometriosis often endure years of suffering before receiving a correct diagnosis [67]. Similarly, women with cardiac chest pain are more likely to be diagnosed with anxiety than heart disease, resulting in delays in critical care [93].

The dismissal of women’s pain may lead to prolonged suffering, reduced quality of life, and increased prevalence of mental health disorders.

### 5.2. Under-Representation in Pain Research and Its Implications

Historically, women have been under-represented in clinical trials, leading to a lack of sex-specific pain treatment strategies. As a result, many pain management guidelines are based on male physiology, overlooking hormonal and metabolic variations that influence pain processing [23,24].

### 5.3. Gendered Bias in Prescribing Patterns and Pain Management

There is a noticeable difference in how pain medications are prescribed. Women, despite reporting higher pain levels, are more often given sedatives or antidepressants rather than appropriate analgesics [1,3,91]. Postoperative studies show that women also receive lower doses of opioids, which may lead to insufficient pain control and prolonged suffering [9,37,94,95].

The issue is further complicated by racial disparities. Black women are 40% less likely to receive pain medication compared to white women for the equivalent medical conditions [39,96]. Similarly, Hispanic women and low-income individuals face significant obstacles to pain management due to systemic healthcare inequities and provider bias [97].

### 5.4. Gender Differences in Pain Medication Metabolism

Biological differences between men and women further complicate pain treatment disparities, as variations in enzyme activity, body fat composition, and hormone interactions influence both the effectiveness and side effects of analgesic treatments. Namely, women tend to exhibit stronger analgesic effects from opioids but are also more likely to suffer from side effects such as nausea and respiratory depression, indicating greater opioid sensitivity [1]. Consequently, they may have a lower likelihood of achieving effective pain relief with opioids and therefore experience greater physical impairment [98].

In addition, women are more prone to gastrointestinal side effects from nonsteroidal anti-inflammatory drugs (NSAID), often requiring alternative treatments such as COX-2 inhibitors or nerve blocks to achieve effective pain control [2]. These sex-based differences underscore the importance of personalized pain management strategies that account for biological and pharmacokinetic variations to optimize treatment efficacy and safety in women.

### 5.5. Psychological and Sociocultural Factors

Societal attitudes toward pain expression differ based on gender. While men are expected to hide their pain, women are encouraged to express it, yet their pain is often not taken seriously [2]. This contradiction affects self-reported pain experiences as well as medical responses, potentially leading to treatment biases.

Gender role expectations also influence how individuals perceive and experience pain. For example, young men tend to view themselves as more tolerant to pain than both the average man or woman, while the typical man is expected to report less pain than the typical woman [22]. Sociocultural expectations further affect racial and socioeconomic groups differently. False beliefs about biological differences between Black and White individuals can lead to racial disparities in pain assessment and treatment recommendations [39]. In addition, financial barriers often prevent low-income women from seeking medical care, potentially exacerbating chronic pain conditions [86].

Women with chronic pain are also more likely to receive diagnoses of depression or anxiety, and their pain is frequently attributed to psychological distress rather than addressed as a physical condition [91]. This may result in inadequate pain management and may increase mental health burden. However, antidepressants may have a beneficial effect by reducing harmful neuroplastic changes in the central nervous system in patients with depression and chronic pain, potentially leading to pain relief [88].

Furthermore, racial differences also play a role in how psychological symptoms are reported. Asian Americans consistently report anxiety at a lower frequency compared to individuals from other racial groups, whereas European Americans report the highest rates of anxiety [99].

## 6. Treatment Approaches for Women’s Pain Management

Effective pain management in women requires a multifaceted approach that considers biological, psychological, and sociocultural influences. This chapter explores pharmacological, non-pharmacological, and integrative therapies aimed at optimizing pain relief in women (Table 1).

### 6.1. Pharmacological Treatments

#### 6.1.1. Analgesics and Anti-Inflammatory Drugs

NSAIDs such as ibuprofen and naproxen are widely used to manage pain conditions, including dysmenorrhea. Research indicates that NSAIDs are more effectful than paracetamol [100]. However, long-term use may lead to gastrointestinal and cardiovascular risks, which necessitate careful monitoring [101].

#### 6.1.2. Opioids

Opioid analgesics may be indicated for severe pain conditions, such as post-surgical pain or chronic pelvic pain. Women generally tend to exhibit greater opioid sensitivity but also higher risks of developing opioid-induced side effects, including nausea, respiratory depression, and addiction [1]. Therefore, risk assessment and individualized dosing are essential for safe opioid use in women.

#### 6.1.3. Hormonal and Adjuvant Analgesics

Hormonal therapies, including oral contraceptives, gonadotropin-releasing hormone (GnRH) agonists, and progestins play a significant role in managing pain related to menstrual disorders and endometriosis [67]. Neuromodulator drugs such as gabapentin and antidepressants such as duloxetine are effective in treating neuropathic pain conditions like fibromyalgia [88,102,103]. In the setting of acute pain, as in the perioperative setting, a multimodal approach with adjuvant analgesics should be implemented [94,104,105] This may include magnesium, ketamine for opioid-dependent patients, and alpha2-agonists [37].

### 6.2. Pain Management Across Different Life Stages and Conditions

#### 6.2.1. Pain Management in the Menstrual Cycle

Hormonal contraceptives can impact pain perception, with some studies indicating that combined oral contraceptives may help menstrual-related pain by stabilizing hormone fluctuations. However, they may increase migraine frequency in susceptible individuals [106]. Hormonal treatments, particularly estrogen therapy, have been explored as preventive options for migraine, although progesterone therapy has shown limited efficacy [107].

#### 6.2.2. Pain Management in Endometriosis

Hormonal therapies, such as GnRH agonists, oral contraceptives, and progestins, are standard treatments for endometriosis but may be ineffective in progesterone-resistant patients. These treatments carry risks, including bone density loss and mood disturbances, necessitating personalized treatment approaches [67]. Emerging therapies include immune modulation, nerve-targeted approaches, cognitive behavioral therapy (CBT), dietary modifications, and neuromodulation, highlighting the need for multimodal, individualized management. In women with pain resistant to conventional therapies, surgical intervention should be evaluated [67].

#### 6.2.3. Pain Management in Pregnancy and for Breastfeeding Women

Due to altered pharmacokinetics, pain relief strategies during pregnancy must balance efficacy and fetal safety. NSAIDs are generally avoided in the third trimester due to risks such as premature closure of the ductus arteriosus [10]. Similarly, opioids must be used cautiously to reduce neonatal opioid withdrawal syndrome. Paracetamol is the most used analgesic in pregnancy, with no studies showing congenital side effects [10]. Non-pharmacological approaches, including physical therapy, are often prioritized for pain relief during pregnancy [10,108,109].

Both pharmacological and non-pharmacological methods are crucial in labor pain management. Epidural anesthesia remains the most effective pain relief option. Supportive therapies such as Pilates, chiropractic care, and music therapy further aid in managing pain during labor [108,109,110].

When medication is necessary in breastfeeding women, paracetamol and ibuprofen are considered safe. Opioids should be used with caution due to potential risks, including neonatal sedation [111].

#### 6.2.4. Pain Management in Menopause

Chronic pain conditions, such as osteoarthritis and osteoporosis-related fractures, become more prevalent after menopause. The primary pharmacological treatment is hormone therapy (either estrogen or estrogen/progesterone combination therapy) (Table 2) [69]. Thereby, natural and synthetic hormones are frequently grouped together, despite substantial differences in their pharmacodynamics and clinical effects [112]. Endogenous or bioidentical estrogens (e.g., 17β-estradiol) differ markedly from synthetic estrogens (e.g., ethinylestradiol) commonly used in oral contraceptives [113]. Similarly, natural progesterone exhibits distinct properties compared to synthetic progestins such as medroxyprogesterone acetate [114]. Route of administration further impacts hormone metabolism and systemic action; for instance, transdermal estradiol bypasses hepatic first-pass metabolism, allowing for more stable plasma concentrations and reduced systemic side effects [115]. These distinctions are clinically relevant, as hormone bioavailability and receptor binding profiles can meaningfully influence pain modulation, inflammatory responses, and overall treatment outcomes [116].

Effective management requires multimodal strategies, including manual therapy [117], magnesium supplementation for musculoskeletal pain [118], and lifestyle modifications [69].

#### 6.2.5. Non-Pharmacological Therapies

Many women benefit from non-pharmacological approaches that address the biopsychosocial aspects of pain. These interventions are particularly useful for chronic pain conditions. Therapeutic exercise, physiotherapy, and stretching programs improve mobility and reduce pain intensity in conditions such as chronic pelvic pain [119], fibromyalgia [120], and osteoarthritis [69].

Psychological factors strongly influence pain perception. Cognitive behavioral therapy (CBT) effectively reduces pain-related distress and enhances coping strategies [121,122]. Mindfulness-based stress reduction (MBSR) has shown benefits in reducing pain severity and improving emotional regulation [122,123]. Antidepressants may also be effective in reducing pain severity and improving quality of life [121].

Acupuncture may be effective for the treatment of chronic pain across different conditions, with treatment effects often persisting over time [124]. Other complementary treatments, such as chiropractic and tai chi, show benefits for pain management [19].

In summary, given the complex nature of pain in women, a multidisciplinary approach that integrates pharmacological and non-pharmacological treatments is often the most effective strategy [19].

## 7. Future Directions in Women’s Pain Management

Despite notable progress, significant gaps in sex-specific pain research and treatment persist. Addressing these gaps requires a focus on inclusive research, equitable treatment, and necessary policy changes. Women’s pain is often mismanaged due to gender bias. Considering hormonal differences in treatment protocols may improve treatment efficacy and reduce side effects. Research must focus on how men and women experience pain differently to develop better treatment strategies. Fair prescribing practices and provider training are essential for reducing disparities in daily care. Personalized treatment approaches—including new therapies such as neuroimmune treatments or estrogen receptor modulator—hold promise in improving pain relief. Studying pain across different life stages and combining medication with non-drug treatments may further improve women’s outcomes. Tackling these disparities requires a multidimensional approach, including personalized pain management strategies, increased awareness of gender biases in medical practice, and expanded research into sex-specific treatment protocols [125,126,127]. Bridging these research gaps is crucial for advancing pain care and overall health improvements [24,128].

In conclusion, women’s pain management is influenced by a complex interplay of hormonal fluctuations, life stages, such as pregnancy and menopause, and psychosocial factors. Despite clear differences in pain perception and response to treatment, current pain management strategies remain predominantly based on male-centric models. This often leads to unequal and less effective care in women.

By tailoring pain management to the specific needs of women, healthcare professionals can improve both pain relief and overall quality of life for women, leading to more equitable and effective healthcare outcomes.

## Figures and Tables

**Table 1:** Summary of hormonal profiles, pain trends, and interventions across the female lifespan.

Life Stage	Hormonal Profile	Common Pain Conditions	Pain Sensitivity Trend	Suggested Interventions
Adolescence	Onset of estrogen/progesterone cycles	Dysmenorrhea, menstrual migraines	Fluctuating; often heightened	Non-steroidal anti-inflammatory drugs, hormonal regulation (e.g., oral contraceptive pills), cognitive behavioral therapy, lifestyle education
Reproductive	Cyclical estrogen/progesterone levels	Endometriosis, fibromyalgia, migraines	Variable (cycle-dependent)	Hormonal therapy (oral contraceptive pills, gonadotropin-releasing hormone analogs), physical therapy
Perimenopause	Declining estrogen, irregular cycles	Premenstrual syndrome, joint pain, mood-associated pain	Increased variability	Multimodal pain management, magnesium supplementation, cognitive behavioral therapy, mindfulness-based stress reduction
Postmenopause	Low estrogen/progesterone	Osteoarthritis, osteoporosis, chronic back pain	Elevated baseline sensitivity	Hormone replacement therapy (individualized), resistance training, non-opioid pain relievers

**Table 2:** Comparison of commonly used estrogens and progestogens.

Hormone	Source	Route	Clinical Notes
17β-Estradiol	Bioidentical	Transdermal/Oral	More physiologic; transdermal route provides stable plasma levels and lower thrombotic risk
Ethinylestradiol	Synthetic	Oral	Requires hepatic metabolism; commonly used in combined oral contraceptives
Micronized Progesterone	Bioidentical	Oral/Vaginal	Exhibits neuroprotective effects; generally well tolerated
Medroxyprogesterone	Synthetic progestin	Oral/Injectable	Associated with more side effects; lacks some of the beneficial effects of bioidentical progesterone

## Data Availability

Not applicable.
